# Identification of Roles for Peptide: *N*-Glycanase and Endo-β-*N*-Acetylglucosaminidase (Engase1p) during Protein *N*-Glycosylation in Human HepG2 Cells

**DOI:** 10.1371/journal.pone.0011734

**Published:** 2010-07-23

**Authors:** Isabelle Chantret, Magali Fasseu, Karim Zaoui, Christiane Le Bizec, Hassane Sadou Yayé, Thierry Dupré, Stuart E. H. Moore

**Affiliations:** 1 INSERM, U773, Centre de Recherche Bichat Beaujon, Paris, France; Université Paris 7 Denis Diderot, site Bichat, Paris, France; 2 Biochimie Métabolique et Cellulaire, Hôpital Bichat-Claude Bernard, AP HP, Paris, France; Massachusetts Institute of Technology, United States of America

## Abstract

**Background:**

During mammalian protein *N*-glycosylation, 20% of all dolichol-linked oligosaccharides (LLO) appear as free oligosaccharides (fOS) bearing the di-*N*-acetylchitobiose (fOSGN2), or a single *N*-acetylglucosamine (fOSGN), moiety at their reducing termini. After sequential trimming by cytosolic endo β-*N*-acetylglucosaminidase (ENGase) and Man2c1 mannosidase, cytosolic fOS are transported into lysosomes. Why mammalian cells generate such large quantities of fOS remains unexplored, but fOSGN2 could be liberated from LLO by oligosaccharyltransferase, or from glycoproteins by NGLY1-encoded Peptide-*N*-Glycanase (PNGase). Also, in addition to converting fOSGN2 to fOSGN, the ENGASE-encoded cytosolic ENGase of poorly defined function could potentially deglycosylate glycoproteins. Here, the roles of Ngly1p and Engase1p during fOS metabolism were investigated in HepG2 cells.

**Methods/Principal Findings:**

During metabolic radiolabeling and chase incubations, RNAi-mediated Engase1p down regulation delays fOSGN2-to-fOSGN conversion, and it is shown that Engase1p and Man2c1p are necessary for efficient clearance of cytosolic fOS into lysosomes. *Saccharomyces cerevisiae* does not possess ENGase activity and expression of human Engase1p in the *png1Δ* deletion mutant, in which fOS are reduced by over 98%, partially restored fOS generation. In metabolically radiolabeled HepG2 cells evidence was obtained for a small but significant Engase1p-mediated generation of fOS in 1 h chase but not 30 min pulse incubations. Ngly1p down regulation revealed an Ngly1p-independent fOSGN2 pool comprising mainly Man_8_GlcNAc_2_, corresponding to ∼70% of total fOS, and an Ngly1p-dependent fOSGN2 pool enriched in Glc_1_Man_9_GlcNAc_2_ and Man_9_GlcNAc_2_ that corresponds to ∼30% of total fOS.

**Conclusions/Significance:**

As the generation of the bulk of fOS is unaffected by co-down regulation of Ngly1p and Engase1p, alternative quantitatively important mechanisms must underlie the liberation of these fOS from either LLO or glycoproteins during protein *N*-glycosylation. The fully mannosylated structures that occur in the Ngly1p-dependent fOSGN2 pool indicate an ERAD process that does not require *N*-glycan trimming.

## Introduction

During protein *N*-glycosylation, oligosaccharyltransferase (OST) transfers the oligosaccharide Glc_3_Man_9_GlcNAc_2_ from the mature lipid linked oligosaccharide precursor (LLO, Glc_3_Man_9_GlcNAc_2_-PP-dolichol) onto asparagine residues in the consensus sequence Asn-X-Ser/Thr of nascent proteins [1]. *N*-linked oligosaccharides play crucial roles in the quality control, folding, ER-associated degradation (ERAD) and subcellular trafficking of glycoproteins [2]. During both mammalian LLO biosynthesis and ERAD, free oligosaccharides (fOS) possessing the di-*N*-acetylchitobiose moiety at their reducing end (fOSGN2), are generated [3], [4]. Evidence suggests that, *in vitro*, fOSGN2 can be released from LLO by OST [5], [6], [7], or from misfolded glycoproteins by cytosolic peptide *N*-glycanase (PNGase) [8]. Accordingly characterising fOS production in cells reveals insights into key regulatory points in protein *N*-glycosylation and quality control.

In mammalian cells fOSGN2 are transported out of the ER into the cytosol [9], [10], and subsequently, after cytosolic trimming [11] are transported into lysosomes [12] to be degraded. Cytosolic trimming of fOSGN2 is accomplished by an endo-β-*N*-acetylglucosaminidase (ENGase, [13]) or chitobiase [14] to yield fOS bearing a single *N*-acetylglucosamine (GlcNAc) residue at their reducing termini (fOSGN). These structures are the preferred substrates for the cytosolic mannosidase [15], encoded by the MAN2C1 gene [16], [17], that trims Man_9_GlcNAc to generate (Manα1-2Manα1-2Manα1-3(Manα1-6)Manβ1-4GlcNAc: linear isomer of Man_5_GlcNAc) that is transported into lysosomes [12].

Although the above outlined scheme for the generation and disposal of fOS has been proposed [18], [19], many aspects of fOS metabolism remain to be elucidated. First, in mammalian cells grown under normal culture conditions up to 20% of all oligosaccharides synthesized as LLO appear as fOS during or rapidly after glycoprotein biosynthesis, but presently the proportion of fOS that are generated from glycoproteins during ERAD or from LLO is unknown. In *S. cerevisiae* the PNG1-encoded cytosolic PNGase [20] generates greater than 70% of all fOSGN2 [3], [21]. By contrast to the situation in yeast, there are no data concerning the importance of Ngly1p [22], the mammalian ortholog of Png1p, during mammalian fOS generation. Second, although *S. cerevisiae* does not possess ENGase activity and all fOS in this organism are generated as fOSGN2, several studies using mammalian cells report circumstantial evidence suggesting that fOSGN may be released directly from glycoproteins by an ENGase [5], [23], [24], [25]. Furthermore, the cytosolic ENGase encoded by the *C. elegans* [26], chicken [27] and human [28] ENGASE gene is capable of generating fOSGN from glycoproteins as well as converting fOSGN2 to fOSGN *in vitro*. However, the *in vivo* roles of mammalian Engase1p [28], during fOS metabolism have yet to be addressed.

Here, we have used RNA interference (RNAi) and pharmacological approaches to knock down Ngly1p and Engase1p activities in order to gain insight into the mechanisms underlying fOS release in the HepG2 cell line. We have characterised and quantitated Ngly1p-dependent and -independent fOSGN2 pools. It is shown that hEngase1p is able to deglycosylate misfolded glycoproteins in an *S. cerevisiae png1Δ* strain and evidence was obtained for a deglycosylating function for this enzyme in HepG2 cells. Results demonstrate that Engase1p plays a major role in the clearance of cytosolic fOS into lysosomes. Finally, it is shown that in HepG2 cells a major fraction of fOS is not generated by either Ngly1p or Engase1p.

## Materials and Methods

### Reagents

HepG2 cells were obtained from ATCC (Rockville, MD). d-mannitol and d-sorbitol were from Fluka (St Quentin Fallavier, France). d-[2-^3^H]mannose (20 Ci/mmole) and En^3^hance spray were purchased from PerkinElmer Life Sciences (Zaventem, BE). Thin Layer Chromatography (TLC) plates were obtained from MERCK (Darmstadt, DE). AG 50-X2 (H^+^ form) and AG 1-X2 (acetate form) came from Biorad S.A. (Marnes la Coquette, FR). Streptolysin O (SLO) was a generous gift from Sucharit Bhakdi (Institute of Medical Microbiology and Hygiene, Mainz, DE). Concanamycin A (CCMA) was a gift from Dr. J.R. Green (Ciba-Geigy, Ltd, CH). Swainsonine (SW), 2-aminopyridine, *N*-benzoyl-L-tyrosine-p-nitroanilide, dimethylformamide, lyticase, reduced glutathione, *p*-nitrophenyl-α-d-glucopyranoside, fucose, sodium cyanoborohydride, Triton ×100, protease inhibitor cocktails, endo-β-*N*-acetylglucosaminidase from *Streptococcus plicatus* (EndoH), brefeldin A (BFA), 3-methyladenine, Kodak X-OMAT AR film and pronase were purchased from SIGMA–Aldrich SARL (St Quentin Fallavier, FR). Methyl α-d-mannopyranoside, methyl-α-d-glucopyranoside and castanospermine (CST) were from Toronto Research Chemicals Inc. (Toronto, CA). Stealth small interfering RNA (siRNA) duplexes, stealth RNAi negative medium and low GC control duplex, reduced serum medium (Opti-MEM I), Lipofectamine RNAiMAX, and the Superscript™ Preamplification System were from Invitrogen (Cergy Pontoise, FR). RNeasy Mini Kit, RNase-free DNase I, the anti-His Antibody Selector Kit and the 6xHis Protein Ladder were obtained from Qiagen (Courtaboeuf, FR). ABsolute Blue QPCR SYBR Green Mix and BCA™ Protein Assay kit were purchased from Thermo Scientific (Courtaboeuf, FR). Yeast minimal SD base medium and minimal SD agar base were purchased from Ozyme (Saint-Quentin-en-Yvelines, FR). The yeast strain BY4742 and Y12156 were obtained from Euroscarf (Frankfurt, DE). The lactate dehydrogenase (LDH) detection cytotoxicity kit was purchased from Roche Diagnostics (Meylan, FR). Z-vad-fmk was from Promega (Charbonnières-les-Bains, FR).

### Cell Culture and transfection

HepG2 cells were routinely cultivated in RPMI 1640 supplemented with 10% foetal calf serum (FCS) and 1% penicillin/streptomycin and were maintained at 37°C in a humidified atmosphere with 5% CO_2_. Sequences of Stealth siRNA duplexes used for specific down regulation of the ENGASE, MAN2C1 and NGLY1 mRNA are listed in Table S1. According to their GC content, medium GC or low GC Stealth RNAi duplexes were used as negative control. All siRNA duplexes were transiently transfected into HepG2 cells by using Lipofectamine RNAiMax according to the protocol of the manufacturer.

### mRNA expression analysis

Total RNA was isolated from transfected HepG2 cells using the RNeasy Mini Kit using an RNase-free DNase I step according to the instructions of the manufacturer. The quality of the total RNA was assessed after agarose gel electrophoresis. First strand cDNA was synthesized from 2.5 µg of total RNA employing the Invitrogen Superscript™ Preamplification System and an oligo(dT)_12-18_ primer as reverse primer. The sequences of the primers used for PCR, real time PCR or sequencing are listed in Table S1. Real time quantification of transcripts was performed using a CHROMO IV Detector (MJ Research, Boston MA) with SYBR Green PCR master mix. All specific primer couples used for real time quantitative PCR (QPCR) were designed to cover exonic sequences separated by large intronic regions. The results of each gene were normalized with the Ct value of β2 microglobulin (β2M) or S14.

### Radiolabeling and permeabilization of cells

48 h after transfection in 6 well plates, cells were pulse-radiolabeled with 20 µCi [2-^3^H]mannose in 500 µl of glucose-free RPMI 1640 medium supplemented with 2% dialyzed FCS, 0.5 mM glucose and 2 mM fucose. For chase conditions, cells were incubated in 1 ml of pre-warmed complete RPMI 1640 medium supplemented with 2 mM fucose; after the indicated chase time, the medium was removed and cells were cooled to 4°C and washed twice with ice cold PBS. Cells were permeabilized with SLO as described previously [11]: cells were incubated at 4°C with 0.5 ml of a pre-cooled permeabilization buffer (5 mM Hepes, pH 7.0, containing 250 mM mannitol and 2 mM CaCl_2_) containing 2 µg/ml of SLO. After 1 h of incubation, the SLO-containing medium was removed from the cells and combined with a subsequent wash of 1 ml permeabilization buffer.

### Recovery of fOS, glycoproteins and LLO from radiolabeled cells

These procedures have been described previously [11], [29]. Briefly the cell layer was scraped into 4 ml of MeOH/100 mM Tris HCl (pH 7.4) containing 4 mM MgCl_2_, 2∶1. An equal volume of CHCl_3_ was added and the mixture vigorously shaken. After centrifugation, the lower (CHCl_3_) and upper (methanolic) phases were removed. Neutral fOS were recovered from the upper methanolic phase whereas LLO were recovered from the lower CHCl_3_ phase and from CHCl_3_/MeOH/H_2_O 10∶10∶3 extracts of the interphase proteins. Oligosacharides were released from LLO after mild acid hydrolysis with 0.02N HCl, 30 min at 100°C. Glycoproteins (GP) from the 10∶10∶3-extracted protein pellet and the TCA-precipitated glycoproteins recovered from cell culture medium were submitted to pronase digestion to yield glycopeptides. Oligosaccharides were released from glycopeptides using EndoH. Recovery of unlabelled fOS from yeast cells, their derivatisation with the fluorophore 2-aminopyridine, and analysis by HPLC will be described elsewhere (Chantret, I. et al., manuscript in preparation).

### Analytical procedures

Glycopeptides were analysed by concanavalin A (ConA)-Sepharose chromatography as described previously [30]. Separation of fOSGN2 and fOSGN was achieved after derivatisation of oligosaccharide mixtures with 2-aminopyridine (AP) as previously reported [31], [32]. All oligosaccharide-AP derivatives are positively charged but only fOSGN2-AP are cleaved after digestion with EndoH, and after AG-50 ion exchange chromatography are recovered as fOSGN in the water wash. The ion-exchange resin is then washed with 0.2 M NH_4_OH in order to elute fOSGN-AP. Oligosaccharides and oligosaccharide-AP derivatives were resolved by thin-layer chromatography (TLC) on silica-coated plastic sheets (0.2 mm thickness) in *n*-propanol/acetic acid/water, 3/3/2 for 16–24h. Radioactive components were detected on X-OMAT AR film by fluorography after spraying the dried TLC plates with En^3^hance and were quantitated by scintillation counting after their elution with water from the silica. Where indicated, fOS mixtures and fOS-AP mixtures were resolved by HPLC using an amine-bonded silica column (LiChrospher Amino 5 µm, 250 mm×4.6 mm, Sulpelco Inc). Two eluents were used: eluent A (90% acetonitrile, 10% 30 mM triethylamine acetate, pH 7.3, buffer) and B (10% acetonitrile, 90% 30 mM triethylamine acetate, pH 7.3, buffer). The column was equilibrated in 85% A and 15% B, and after sample injection, was subjected to a linear solvent gradient developed over 80 min until the final solvent mixture of 68% of A and 32% of B was obtained. Radiolabeled oligosaccharides were detected by a Packard 150 TR flow-scintillation analyser and oligosaccharide-AP derivatives were quantitated using a Jasco FP-2020 Plus fluorescence detector (excitation wavelength 310 nm, emission wavelength 380 nm).

### Yeast strains, plasmid construction, transformation and culture

*Saccharomyces cerevisiae* strains used in this study are: BY4742 (*MATa*; *his3Δ1*; *leu2Δ0*; *lys2Δ0*; *ura3Δ0*), Y12156 (*MATa*; *his3Δ1*; *leu2Δ0*; *lys2Δ0*; *ura3Δ0*; *png1::kanMX4*), *ams1Δ* (*MATa*; *ams1*::*HIST3MX6*; *leu2Δ0*; *lys2Δ0*; *ura3Δ0*) and *ams1Δpng1Δ* (*MATa*; *ams1::HIST3MX6*; *leu2Δ0*; *lys2Δ0*; *ura3Δ0*; *png1::kanMX4*). The last two strains were derived from BY4742 and Y12156 respectively and were generated by homologous recombination [33] using the PCR product generated with the template pFA6a-His3MX6 plasmid kindly provided by M. S. Longtine (Oklahoma State University, USA). Yeast were transformed using lithium acetate, and transformants were isolated after growth on solid selective medium (lacking histidine). The correct replacement of the target gene by the auxotrophic His3MX6 marker, was confirmed by PCR amplification. A plasmid encoding His_6_-tagged Engase1p was kindly provided by T. Suzuki (RIKEN Advanced Science Institute, JP). The coding region of human ENGASE was amplified by PCR: the his_6_-tag sequence was added in-frame immediately before the stop codon. After digestion with XbaI and SalI, the PCR product was subcloned into the URA3 pRS416-GPD plasmid. The correct sequence of the insert was verified by DNA sequencing. Yeasts were transformed as above with either the plasmid construct or the empty vector. Culture of transformants was performed in liquid selective medium (lacking uracil).

### Yeast subcellular fractionation and enzyme assays

Cells were harvested by centrifugation and washed with distilled water at room temperature. The pellet was then resuspended in 1 ml of buffer A (50 mM Tris/HCl pH 8, 1.5 M sorbitol, 2 mM MgCl_2_, 60 mM β-mercaptoethanol). After 10 min of preincubation at 30°C, cells were converted to spheroplasts by adding lyticase (10 units/OD_600nm_ yeast) and incubating 15 min under gentle shaking at 30°C. Spheroplasts were loaded onto a cushion of 500 µl of buffer B (50 mM Tris/HCl pH 8, 1.8 M sorbitol, 2 mM MgCl_2_) in an Eppendorf tube kept on ice. After centrifugation for 5 min at 6000 gAv at 4°C, the pellet is carefully resuspended in buffer A. After repeating this procedure twice, the pellet is resuspended in buffer C (20 mM K^+^/Pipes, pH 6.8, 100 mM sorbitol, 100 mM KCl, 50 mM K^+^/acetate, 5 mM magnesium acetate) and spheroplast permeabilization based on differential osmotic lysis was performed as previously described [34] for 30 min on ice. After centrifugation for 10 min at 20,000 gAv, the supernatant containing cytosol, and the pellet were separated and the membrane fraction was homogenized in buffer C. Yeast α-glucosidase (αGlc) [35] and Carboxypeptidase Y (CPY) [36] were measured as previously described. For ENGase assay, [2-^3^H]-mannose labeled polymannose-type glycopeptides were incubated overnight at 37°C in the presence of aliquots of subcellular fractions derived from mock transformed or hEngase1p-expressing yeast. The reactions were heated for 5 min at 100°C prior to centrifugation for 5 min at 10000 gAv. The supernatant was loaded on AG-1/AG-50 column. The effluent and water washes contain neutral oligosaccharides which were quantitated by scintillation counting. Background radioactivity observed for fractions derived from mock transformed yeast were subtracted from that observed in fractions from the hEngase1p expressing strain.

For SDS-PAGE, yeast cells were harvested at mid-log growth, washed, and after adding the same volume of glass beads, were extracted with lysis buffer (50 mM Tris/HCl, pH 8.0, 2 mM PMSF supplemented with protease inhibitors). The cells were disrupted via three sequential 60 s burst on a Vortex mixer followed by cooling on ice for 60 s. After 5 min heating at 95°C, the resulting supernatant was cleared by centrifugation at 10000 gAv for 10 min. SDS-PAGE was followed by immunoblotting with the tetra-His antibody.

## Results

### Experimental strategy

Potentially both Ngly1p and Engase1p could give rise to fOS in mammalian cells and could have overlapping functions. Accordingly, inhibition of one enzyme may lead to compensatory activity by the other leading to difficulty in ascertaining the normal roles of these enzymes. Thus our strategy was to look at the effects of inhibiting the enzymes either separately or together. We started with down regulating Engase1p as this enzyme is potentially involved in both glycoprotein deglycosylation and in the conversion of fOSGN2 to fOSGN. The ability to block this latter reaction would greatly facilitate interpretation of experiments where the role of Ngly1p in generating fOSGN2 is evaluated.

### Engase1p down regulation slows down fOSGN2-to-fOSGN conversion in HepG2 cells

**Figure 1:**
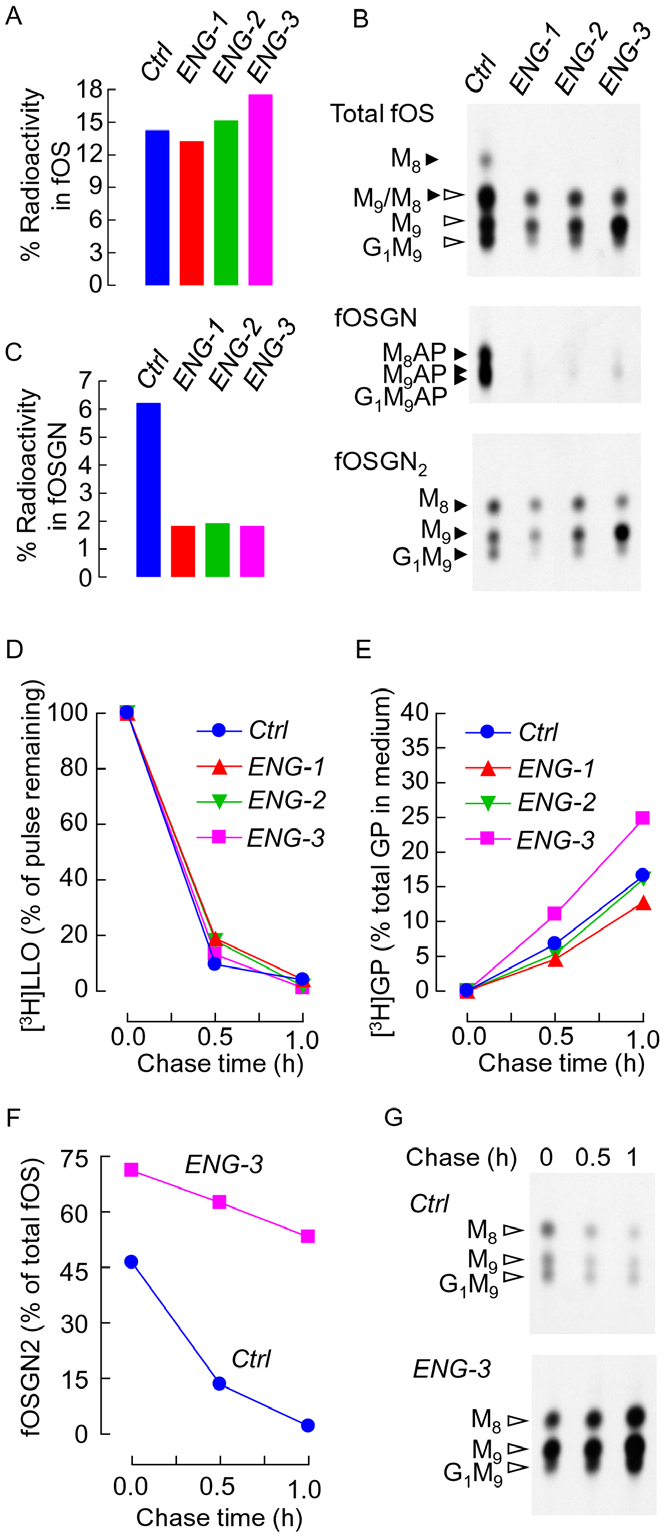
ENGASE mRNA silencing alters fOS metabolism. A. Three days post transfection with 25 pmoles of control RNAi duplexes (*Ctrl*) or ENGASE RNAi duplexes (*ENG-1*, *ENG-2* and *ENG-3*), cells were pulse-radiolabeled with [2-^3^H]mannose for 30 min. Incorporation of [2-^3^H]mannose into LLO, Glycoproteins and fOS was measured and summed to give total cellular radioactivity. The percentage of this total occurring as [2-^3^H]fOS was calculated. B. [2-^3^H]fOS were resolved by TLC (upper panel). After derivatisation with 2-aminopyridine, EndoH digestion and fractionation of incubation mixtures on AG-50 ion-exchange resin, [2-^3^H]fOS were separated into [2-^3^H]fOSGN (originally fOSGN2) and [2-^3^H]fOSGN-AP (originally fOSGN) before analysis by TLC. The migration positions of standard oligosaccharides or oligosaccharide AP-derivatives are shown to the left of the chromatographs. Closed and open arrowheads indicate the migration positions of fOSGN and fOSGN2 respectively. The abbreviations are: G_1_M_9_; Glc_1_Man_9_GlcNAc_1-2_, M_9_; Man_9_GlcNAc_1-2_, M_8_; Man_8_GlcNAc_1-2_. C. The percentage of total radioactivity incorporated into LLO glycoproteins and fOS recovered as [2-^3^H]fOSGN was calculated. D-E. Cells transfected with either *ENG-1*, *ENG-2*, *ENG-3* or control RNAi duplexes were pulse radiolabeled for 30 min (t = 0) and then chased in normal growth medium for 30 min and 1 h. D. [^3^H]LLO were recovered from the cells and quantitated by scintillation counting. E. [^3^H]glycoproteins were recovered from the cells and incubation media and the percentage of total [^3^H]glycoproteins (cells+medium) recovered from the media is reported. F-G. Cells transfected with *ENG-3* or control RNAi duplexes were pulse radiolabeled for 30 min and then chased in normal growth medium for 30 min and 1 h. F. After recovery of fOS, the amount of fOS occurring as fOSGN2 was calculated as percentage of total fOS. G. Examination of the fOSGN2 species by TLC. Although the effects of the different RNAi duplexes were noted several times and found to be reproducible, the detailed analyses shown in A,C, and D-G were performed once.

Two human EST sequences possibly derived by alternative splicing of the cytosolic ENGase gene (ENGASE) have been reported: one encodes a protein of 377 amino acids whose function is unknown (Gene bank accession FLJ21865) and the other encodes a 743 amino acid protein (Engase1p) possessing glycopeptide hydrolysing activity *in vitro* (Gene bank accession AJ397822, [28]). Using cDNA generated from HepG2 cell RNA, we could amplify the latter but not the former transcript (data not shown). Accordingly, 3 siRNA duplexes (*ENG-1*, *ENG-2* and *ENG-3*) targeting the transcript encoding the longer protein were designed (Table S1). HepG2 cells were transiently transfected with increasing quantities of either negative control or the ENGASE-targeted siRNA duplexes, and ENGASE mRNA silencing was evaluated by RT-QPCR. As demonstrated in Fig S1, these agents inhibited ENGASE mRNA levels in a dose-dependent manner and *ENG-3* was the most efficient causing a 70% decrease of ENGASE mRNA. To assess the consequence of this mRNA silencing on fOS metabolism, HepG2 cells were radiolabeled with [2-^3^H]mannose for 30 min. All three siRNA duplexes induce a concomitant reduction of incorporation of radiolabel into [2-^3^H]LLO, [2-^3^H]glycoproteins and [2-^3^H]fOS. But, when [2-^3^H]fOS is calculated as a percentage of total [2-^3^H]mannose incorporation into cells ([2-^3^HLLO + [2-^3^H]glycoproteins + [2-^3^H]fOS), ∼15% total cell radioactivity was recovered as [2-^3^H]fOS in both ENGASE-targeted siRNA and control transfected cells (Fig 1A). TLC analysis of fOS from pulse radiolabeled control cells (Fig 1B, upper panel) reveals a mixture of fOSGN2 and fOSGN comprising mainly Glc_1-0_Man_9-8_GlcNAc_2_ and Glc_1-0_Man_9-8_GlcNAc as previously described [11], [32]. By contrast, in HepG2 cells transfected with ENGASE-targeted siRNA duplexes, there is a striking reduction of fOSGN (Fig 1B, upper panel). The proportion of total [2-^3^H]fOS occurring as [2-^3^H]fOSGN and [2-^3^H]fOSGN2 was estimated after derivatization of [2-^3^H]fOS with 2-aminopyridine (2-AP, see Materials and Methods). Using this data the incorporation of radiolabel into fOSGN2 and fOSGN was calculated and expressed as a percentage of total cellular incorporation of radiolabel (Fig 1C). Under these conditions the 3 siRNA duplexes provoke ∼75% inhibition of the appearance of fOSGN (Fig 1C). TLC analysis of the fOSGN and fOSGN-AP species demonstrated that, as expected [32], both fOS populations included components containing 8 or 9 residues of mannose and a monoglucosylated species comprising 9 residues of mannose (Fig 1B, middle and lower panels). The reduction of incorporation of radioactivity into LLO, glycoproteins and fOS provoked by the 3 ENGASE-targeted siRNA duplexes could be due to a general slowing down of the glycosylation pathway leading to an apparent increase in the ratio of fOSGN2 to fOSGN. To investigate this possibility pulse radiolabeling was followed by 30 min and 1 h chase incubations in the presence of complete growth medium. Data shown in Fig 1D demonstrate that the rate of consumption of [^3^H]LLO observed in cells transfected with the 3 ENGASE-targeted siRNA duplexes is not significantly reduced compared to the rate seen in cells transfected with the control RNAi duplex. Furthermore, although some variability was noted, the 3 ENGASE-targeted siRNA duplexes did not have a systematic effect on the rate of glycoprotein secretion from cells when compared to the secretion observed from cells transfected with the control RNAi duplex (Fig 1E). After 1 h chase incubations there are approximately equal amounts of fOSGN2 and fOSGN in *ENG-3*-treated cells whereas in control cells fOS comprise >95% fOSGN (Fig 1F). Also, it was noted that even after a 1 h chase period in *ENG-3*-treated cells, greater than 95% of all fOSGN2 occurred as components co migrating with standard Glc_1_Man_9_GlcNAc_2_, Man_9_GlcNAc_2_, and Man_8_GlcNAc_2_ (Fig 1G). To summarize, down-regulation of ENGASE expression delays clearance of fOSGN2 from cells. In the experiments reported above we were unable to detect a correlation between the efficacities of the three duplexes to inhibit mRNA expression (Fig S1) and their abilities to block the appearance of fOSGN (Fig 1C). Although we do not understand the origin of this discrepency, it was noted that *ENG-1* and *ENG-2* led to the lowest incorporation of [2-^3^H]mannose into glycoconjugates. For this reason all further analysis was performed using *ENG-3*.

### Engase1p trimming of fOSGN2 is required for normal fOS trafficking in HepG2 cells

**Figure 2:**
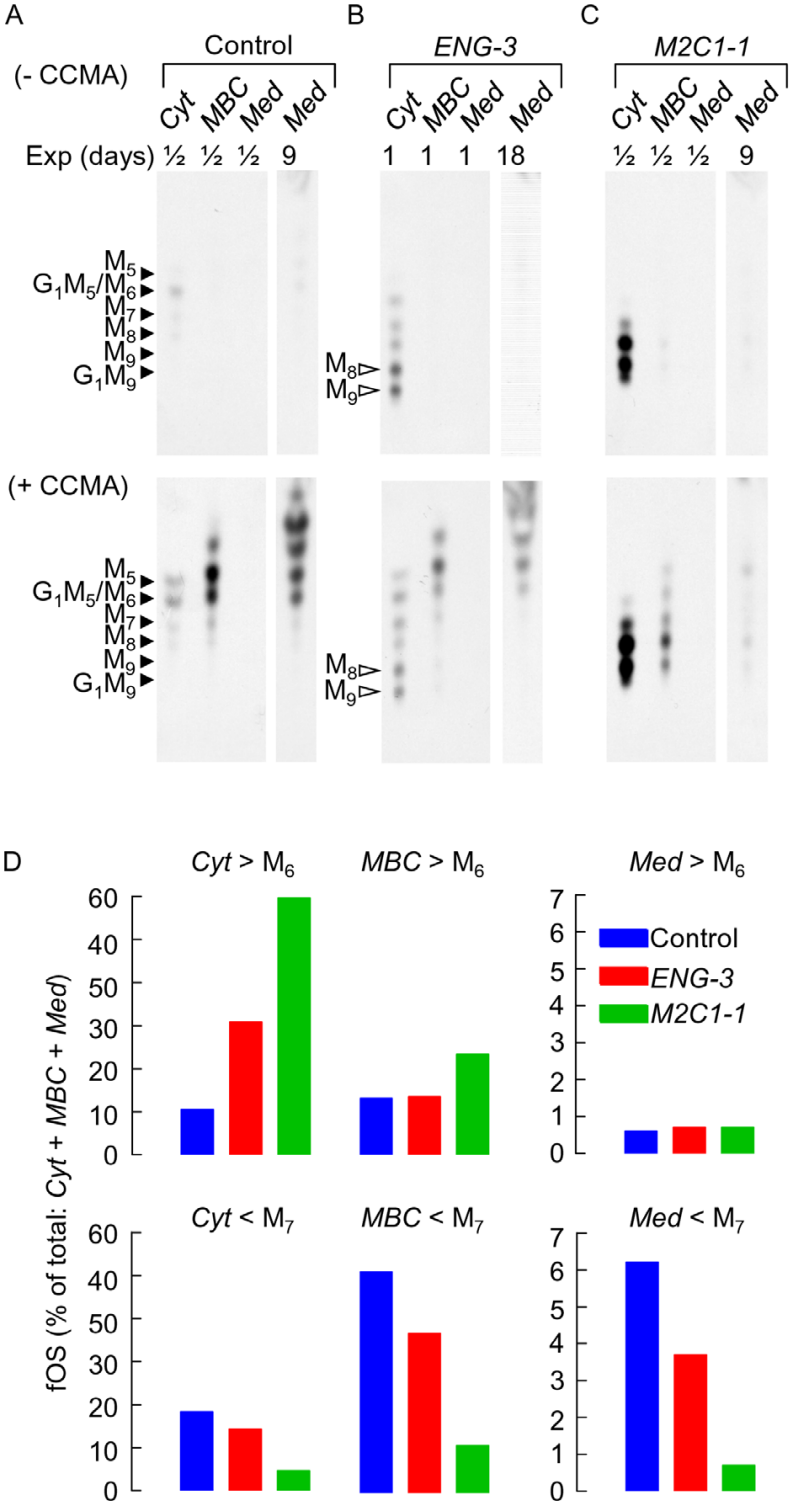
Subcellular distribution of fOS in control and CCMA-treated cells after down regulation of Engase1p and Man2c1p. Three days after cells were transiently transfected with either (A) control RNAi duplexes (Control) or (B) RNAi duplexes targeting ENGASE (*ENG-3*) or (C) MAN2C1 (*M2C1-1*), cells were pulse-radiolabeled with [2-^3^H]mannose, and submitted to 6 h chase incubations in the absence (A, B and C, upper panels) or the presence of 10 nM CCMA (A, B and C, lower panels). Cells were then permeabilized with SLO as described in Material and Methods. fOS purified from the cytosolic fraction (Cyt) and the membrane bound compartments (MBC) and from the chase media (Med) were resolved by TLC. TLC plates were exposed to film for different numbers of days (Exp) in order to better visualise components whose abundances differ greatly between conditions. The migration positions of standard oligosaccharides are shown to the left of the chromatographs and the abbreviations associated with the open arrowheads are: M9, Man_9_GlcNAc_2_; M8, Man_8_GlcNAc_2_; the abbreviations associated with the closed arrowheads are: G1M9, GlcMan_9_GlcNAc; M9, Man_9_GlcNAc; M8, Man_8_GlcNAc; M7, Man_7_GlcNAc; M6, Man_6_GlcNAc; G1M5, GlcMan_5_GlcNAc; and M5, Man_5_GlcNAc. D. Oligosaccharides were eluted from the chromatograms corresponding to the fluorograms shown in the lower panels of A, B and C (+ CCMA) and quantitated by scintillation counting. To simplify interpretation of results, radioactivity associated with oligosaccharides greater in size than Man_6_GlcNAc (> M_6_) was summed as was that obtained for oligosaccharides smaller than Man_7_GlcNAc (< M_7_). The bars indicate the percentage of total fOS recovered from each incubation. This experiment was performed once.

Previous results have indicated that prior to efficient translocation into lysosomes, cytosolic fOSGN are partially demannosylated by a cytosolic mannosidase [11], which corresponds to the protein encoded by the MAN2C1 gene [17]. It is known that this enzyme acts more effectively on fOSGN substrates than their fOSGN2 counterparts [37]. As the results described in Fig 1 demonstrate that Engase1p has an important role in the conversion of fOSGN2 to fOSGN, an experiment was performed in order to evaluate the impact of this block on fOS trafficking in HepG2 cells and compare this effect with that caused by down regulation of Man2c1p. First, 3 MAN2C1-targeted siRNA duplexes (M2C1-1, M2C1-2 and M2C1-3, Table S1) were tested, and 2–3 days after transfection all three were found to decrease MAN2C1 transcript levels by 75–80% without greatly affecting mRNA expression of the Golgi Man2a1p and lysosomal Man2b1p α-mannosidases (Fig S2A). Preliminary experiments in which cells were pulse radiolabeled with [2-^3^H]mannose for 30 min prior to a 4 h chase incubation revealed that all 3 duplexes provoked a striking inhibition of [2-^3^H]fOSGN mannose trimming (Fig S2B) without significantly affecting *N*-glycan trimming in the Golgi apparatus (Fig S2C). Accordingly, cells were transfected with control, ENGASE- (*ENG-3*), and MAN2C1- (*M2C1-1*) targeted siRNA duplexes 2 days prior to performing pulse-chase experiments. After 6 h chase incubations, the cells were permeabilised with streptolysin O (SLO) as previously described to yield a cytosol fraction containing cytoplasmic components including lactate dehydrogenase (LDH), and a residual cellular fraction that has been shown to contain intact lysosomes, Golgi apparatus, and ER (Membrane bound compartments, MBC) [32]. Finally, in order to better evaluate cytosol-to-lysosome translocation of fOS a second series of pulse chase studies were performed in the presence of the vacuolar ATPase inhibitor concanamycin A (CCMA). This agent is known to inhibit the degradation of fOS after their translocation into lysosomes [11] while at the same time blocking lysosomal glycoprotein degradation thereby allowing analysis of fOS metabolism without interference from oligosaccharides potentially derived from the latter process. After 6 h of chase, control incubations (Fig 2A, upper panel) yield low amounts of fOS in the cytosol and MBC fractions, and only trace amounts of these components can be detected in the culture medium (*Med*). By contrast, in the presence of CCMA (Fig 2A, lower panel), striking increases in the quantity of fOS containing 6 or less residues of mannose are observed in the MBC and chase medium. Radioactivity associated with fOS possessing more than 6 residues of mannose and that associated with those possessing less than 7 residues of mannose was assayed in each compartment and expressed as a percentage of radioactivity associated with total cellular and medium fOS (Fig 2D). It can be seen that small fOS recovered from the media of CCMA-treated cells accounts for less than 10% of radioactivity associated with total fOS. *ENG-3* provokes an accumulation of cytosolic fOSGN2 (compare upper and lower panels of Fig 2B with those of Fig 2A) and quantitation of fOS that accumulate in the presence of CCMA (Fig 2D) demonstrates that this agent reduces the appearance of smaller fOS (< Man_7_GlcNAc) in both the MBC and cell medium. Likewise, *M2C1-1* causes a striking accumulation of large fOSGN (> Man_6_GlcNAc) in the cytosol (compare upper and lower panels of Fig 2C with those of Fig 2A), and a concomitant reduction in smaller fOS (< Man_7_GlcNAc) in both the MBC and cell medium (Fig 2D). Accordingly, down-regulation of Engase1p expression slows down the conversion of fOSGN2 into fOSGN and, similarly to down regulation of Man2c1p, reduces the rate at which small fOSGN gain access to the lysosome and extracellular space.

### Down regulation of ENGASE expression modifies the steady state levels of fOS in both the cytosol and MBC of HepG2 cells

**Figure 3:**
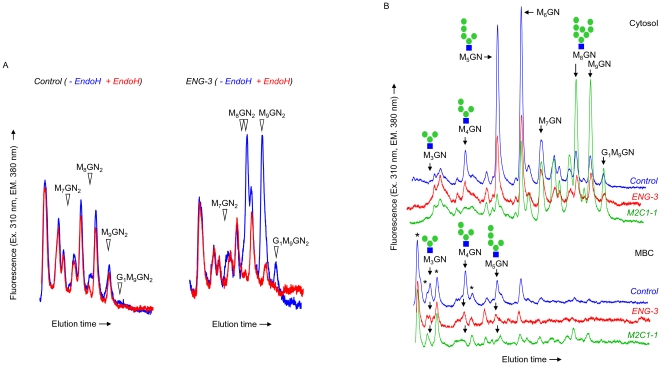
Effects of Engase1p and Man2c1p down regulation on steady-state levels of cytosolic and MBC fOS in HepG2 cells. fOS recovered from both the cytosol and the MBC fractions of SLO-permeabilized, control siRNA (*Control*), *ENG-3* or *M2C1-1* -transfected cells, were derivatized with 2-AP, and analyzed by HPLC before (*− EndoH*) and after digestion with EndoH from *S. plicatus* (*+ EndoH*). A. HPLC profiles of the cytosolic PA-derivatized fOS obtained before (Blue traces) and after EndoH (Red traces) digestion from control siRNA (Left panel) or *ENG-3* (Right panel) transfected HepG2 cells. The open arrowheads indicate the migration positions of fOSGN2-AP derivatives whose appearance is abrogated after EndoH digestion. Only the region of the chromatograms that display significant differences before and after EndoH digestion is shown. B. The EndoH treated fOS from the cytosol (Upper panel) and membrane bound compartments MBC (Lower panel) were resolved by HPLC and the migration positions of standard radioactive oligosaccharide 2-AP derivatives are indicated, and where known, the isomeric configuration of the standard structures are indicated (Green circles; mannose, blue squares; *N*-acetylglucosamine). The asteriks indicate peaks corresponding to oligosaccharides whose abundance does not change under the different conditions. In both A and B the background noise associated with the *ENG-3* profiles is higher than in either the control or *M2C1-3* traces because the fluorescence scale was amplified to take into account the smaller amount of cells recovered from *ENG-3* transfected cultures. This experiment was performed once, but a preliminary experiment, in which cells were not permeabilised with SLO, was performed and found to give qualitatively similar results.

A detailed analysis of the steady state levels of cytosolic fOS in HepG2 cells has been reported, and although fOSGN2 were not detected, a complex mixture of glucosylated and nonglucosylated fOSGN was described [38]. In another report down-regulation of Man2c1p using an siRNA strategy in HEK-293 cells caused an accumulation of the substrates of this enzyme (Glc_1-0_Man_9-7_GlcNAc) but little reduction in its limit digest product (Man_5_GlcNAc) steady state level, indicating an alternative, quantitatively important, mechanism for the generation of this latter component in these cells [17]. In order to further examine the consequences of Engase1p down-regulation on fOS metabolism, fOS steady state levels were examined in the cytosol and MBC of cells transfected with *ENG-3* and compared to fOS levels observed in cells transfected with control and *M2C1-1* siRNA duplexes. Total fOS were extracted from the different compartments and derivatised with 2-AP. The resulting fOS-AP derivatives were then resolved by HPLC before and after digestion with EndoH: only fOSGN2-AP are cleaved and loose their fluorescent tag. It was observed that the EndoH treatment only modified fOS elution profiles in a region of the chromatographs corresponding to the elution times of Glc_1_Man_9_GlcNAc_2_-AP and Man_9-7_GlcNAc_2_-AP standards, and this region of the elution profiles is shown in Fig 3A. Differences in the superimposed HPLC profiles (− EndoH/+ EndoH) as shown in Fig 3A for the cytosol fractions of control-transfected (left panel) and *ENG-3*-transfected (right panel) cells reveal the presence of only small quantities of fOSGN2 in control cells and 6–11 fold increases in these components in *ENG-3*-transfected cells (Table 1). The major species that accumulate in the *ENG-3*-transfected cells appear to elute similarly to standard Glc_1_Man_9_GlcNAc_2_-AP, Man_9_GlcNAc_2_-AP and Man_8_GlcNAc_2_-AP. By contrast to the metabolic radiolabeling experiments shown in Fig 1, small quantities of an oligosaccharide behaving as Man_7_GlcNAc_2_-AP were also noted in the *ENG-3*-transfected cells (Fig 3A, right panel). In addition, the HPLC profiles indicate that the peak corresponding to Man_8_GlcNAc_2_-AP is not symmetrical and comprises a shoulder on its leading edge indicating the presence of a second component (Fig 3A, right panel). At present it is not clear whether this second minor Man_8_GlcNAc_2_-AP component and the Man_7_GlcNAc_2_-AP structure represent slow processing of Glc_1-0_Man_9-8_GlcNAc_2_ oligosaccharides by cytosolic mannosidase or whether they arise directly from either LLO or glycoprotein. In cells transfected with *M2C1-1*, large quantities of interfering fOSGN impeded unambiguous detection of fOSGN2 (results not shown). Finally, with the amount of starting material used, fOSGN2 could not be clearly identified in MBC fractions. Fig 3B shows the chromatographs obtained when the EndoH-treated samples containing only fOSGN-AP were analysed. A detailed analysis of the changes in fOSGN-AP caused by the different siRNA duplexes is beyond the scope of the present report, but two important observations can be underlined. First, as well as their capacity to increase large cytosolic fOS levels (Glc_1-0_Man_9-7_GlcNAc_2_ for *ENG-3*: Fig 3A and Glc_1-0_Man_9-7_GlcNAc for *M2C1-1*: Fig 3B, green trace) *ENG-3* and *M2C1-1* siRNA duplexes reduce the steady state concentration of cytosolic Man_5_GlcNAc, and do so with a relative potency that mirrors their ability to provoke the accumulation of fOSGN2 and untrimmed fOSGN, respectively. Second, data presented in Table 1 indicate that for large fOS, ∼5–15% of the total cellular amount (Cyt + MBC) of each component is associated with the MBC, however this value increases to 50–90% for oligosaccharides that co migrate with standard Man_4-3_GlcNAc. This observation is consistent with previous results indicating a predominantly lysosomal localization of fOSGN possessing less than 5 residues of mannose [11]. Results presented in both Fig 3B and Table 1 demonstrate that in addition to reducing levels of cytosolic Man_5_GlcNAc, both the *ENG-3* and *M2C1-1* siRNA duplexes reduce the levels of Man_4-3_GlcNAc that occur in the MBC. To summarize, the effects of *ENG-3* and *M2C1-1* siRNA duplexes on steady state levels of fOS in HepG2 cells are consistent with the hypothesis that Engase1p and Man2c1p have a concerted action in regulating the subcellular trafficking of fOS in HepG2 cells.

**Table 1:** The effects of MAN2C1- and ENGASE-targeted siRNA duplexes on fOS steady state levels in HepG2 cells.

			*ENG-3*		*M2C1-1*	
Component (% in MBC^c^)			*Fold Ctrl* a (*% in MBC* b)			
fOSGN2^d^	G_1_M_9_		11.1		-e	
	M_9_		6.1		-	
	M_8_		7.0		-	
fOSGN	G_1_M_9_		1.2		6.3	(4.2)
	M_9_	(7.4)	1.2		6.6	(6.3)
	M_8_	(12.4)	1.0		5.1	(8.6)
	M_7_	(10.4)	0.9		1.2	(10.2)
	M_6_	(13.9)	0.5	(9.2)	0.4	(12.0)
	M_5_	(12.6)	0.4	(10.7)	0.1	(5.7)
	M_4_	(53.0)	0.4	(48.9)	0.1	(60.2)
	M_3_	(>90)	0.4	(>90)	0.2	(>90)

a HepG2 cells were transfected with control and ENGASE (*ENG-3*)- and MAN2C1 (*M2C1-1*)-targetted RNAi duplexes prior to SLO permeabilisation to generate cytosol and MBC fractions. After extraction, purification, derivatisation, fOS were resolved by HPLC as described for Fig 3A and B.

b Where peaks were clearly identified, the distribution of each component in the cytosol and MBC fractions after permeabilisation with SLO was calculated as % total component in MBC (% in MBC). Where this value is not shown, low levels of material recovered from the MBC fraction did not permit unambiguous detection of component.

c The calculations described above^b^ were also performed for fOS identified in cells transfected with the control siRNA duplex.

d fOSGN2 were quantitated by HPLC and fluorescence detection of total fOS-AP before and after endoH treatment as described in Fig 3A. HPLC profiles indicate the presence of at least two components migrating similarly to Man_8_GlcNAc_2_-AP (Fig 3A, right panel) and these two components were quantitated together. Small amounts of a component migrating as Man_7_GlcNAc_2_-AP (Fig 3A, right panel) were also identified but were not quantitated.

e Transfection of HepG2 cells with *M2C1-1* provoked large accumulations of fOSGN which masked the appearance of fOSGN2.

### HepG2 cells express two alternatively spliced NGLY1 variants but the effect of Ngly1p on fOS generation is attributable to the protein encoded by the longest transcript

**Figure 4:**
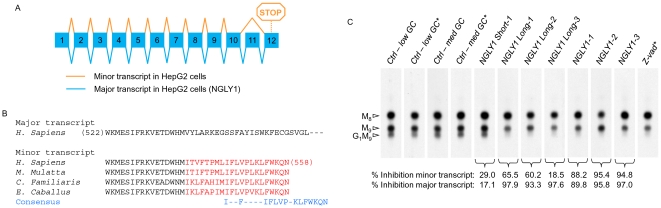
Alternative NGLY1 transcripts and the impact of their down regulation on fOS generation in HepG2 cells. A. Genomic organization of the human NGLY1 gene and alternative transcripts found in HepG2 cells. The major transcript found in HepG2 cells is indicated in blue and corresponds to the already published NGLY1 mRNA sequence (NM_018297.3). A minor transcript indicated in orange is generated by skipping of exon 11 (NM_001145295.1). B. Partial alignment of homologous Ngly1p sequences (amino acids 522–558) encoded by the minor transcript and comparison with the corresponding region of the major NGLY1 transcript (accession numbers: *Macaca mulatta*; XP_001092914, *Equus caballus*; XP_001492093, *Canis lupus familiaris*; DN746692.1). Note that the isoform encoded by the minor transcript contains 21 amino acids in its COOH terminal which are not found in the peptide sequence encoded by the major transcript. C. HepG2 cells were transiently transfected with the different siRNA duplexes designed to silence either or both NGLY1 transcripts (see Table S1). Total RNA were extracted three days later and the mRNA levels of each specific transcript were analyzed by QPCR. In parallel, HepG2 cells were co transfected with *ENG-3* and the different siRNA duplexes targeting the two NGLY1 transcripts. As described in Materials and Methods, because of the variable GC content of the different RNAi duplexes, two negative control siRNA duplexes with either medium (med GC) or low GC (low GC) content were used. After 3 days cells were pulse-radiolabeled with [2-^3^H]mannose for 30 min. In some experiments, cells transfected with *ENG-3* alone or with control RNAi duplexes were treated with either Z-vad-fmk (40 µM) dissolved in DMSO (Z-vad*) or DMSO alone (*) for 45 min prior to, and during the radiolabeling period. Subsequently, [2-^3^H]fOS, [2-^3^H]LLO and [2-^3^H]Glycoproteins were extracted, purified and quantitated, and [2-^3^H]fOS were analysed by TLC. In order to take into account the differences in total incorporation of radiolabel into cells cultivated under the different conditions, a fraction of total fOS was loaded onto the TLC according to the ratio of the total cellular radioactivity for a given incubation to that recovered from the incubation incorporating least radioactivity. The scanned TLC lanes are from the same fluorograph, but due to uneven migration, the scans were aligned manually to facilitate interpretation of data. The migration positions of standard oligosaccharides are shown to the left of the chromatograms and the abbreviations used are as described in Fig 1. The percent inhibition of the major and minor NGLY1 transcripts provoked by the different RNAi duplexes was calculated using the QPCR data and is indicated underneath the appropriate lanes. This experiment was performed once.

The preceeding results indicate that Engase1p has an important role in conversion of fOSGN2 to fOSGN but only a minor, if any, role in glycoprotein deglycosylation. Next, the role of the NGLY1-encoded cytosolic PNGase in fOS generation was examined in HepG2 cells. First, RNAi duplexes that effectively targeted NGLY1 expression were sought, but searches *in silico* indicated several EST sequences potentially arising from alternative transcripts. In order to detect these transcripts, primers were designed to span the regions containing sequence differences but after RT-PCR, only the region corresponding to the C-terminus of Ngly1p yielded different amplified products. Indeed, using forward primer NGLY1-10long and the reverse primer NGLY1-8 (Table S1), the major PCR product (339 bp) expected of the published human NGLY1 mRNA sequence [20] and a minor PCR product of 161 bp were detected. Both PCR products were sequenced and found to correspond to the transcripts shown in Fig 4A. The minor transcript was found to correspond to the EST sequence (Gene bank accession NM_001145295.1) in which exon 11 has been skipped, generating a premature STOP codon that in turn shortens the peptide sequence at its C-terminus. The missing region in this transcript comprises most of the C-terminal domain that is required for the binding of Ngly1p to *N*-linked oligosaccharides [39], suggesting that these two NGLY1 gene products may not have equivalent functions. Furthermore, inspection of the databases revealed the presence of this minor NGLY1 transcript in the sequence data from several mammals (Fig 4B). Next, the impact of these two NGLY1 gene products on fOS generation was examined. siRNA duplexes targeting both common and distinctive regions of the transcripts were designed (Table S1) and the ability of each to silence the two transcripts was assessed by QPCR. Additionally, the ability of the duplexes to block fOSGN2 generation was examined. In order to simplify identification of efficient NGLY1-targeted RNAi duplexes, fOS were examined in a background of Engase1p down regulation where the majority of fOS occur as fOSGN2. Results shown in Fig 4C demonstrate that among the three duplexes which cause greater than 88% silencing of both transcripts, *NGLY1-1* and *NGLY1-3* provoke a striking reduction in the generation of a fOSGN2 migrating as Glc_1_Man_9_GlcNAc_2_, and a lesser reduction of a fOSGN2 migrating as Man_9_GlcNAc_2_, whereas the fOSGN2 migrating as Man_8_GlcNAc_2_ appeared to be unaffected. The fOSGN2 profiles obtained with *NGLY1-1* and *NGLY1-3*, but not *NGLY1-2*, were found to be identical to that obtained when the *ENG-3* transfected cells were pretreated with, and radiolabeled in the presence of the PNGase inhibitor Z-vad-fmk (Z-vad) [40] (Fig 4C). Although successful design of an effective duplex specifically targeting the minor transcript was not achieved (Fig 4C), one of the 3 duplexes targeting the major NGLY1 transcript selectively silenced this transcript without silencing the minor transcript (*NGLY1 Long-3*). The fOS profile obtained with this duplex was similar to those observed with Z-vad and *NGLY1-1* and *NGLY1-3* duplexes, indicating that the major NGLY1 transcript that encodes Ngly1p is responsible for the observed changes in fOS generation in HepG2 cells (Fig 4C).

### siRNA duplexes targeting NGLY1 inhibit the generation of a pool of fOSGN2 comprising mainly Glc_1_Man_9_GlcNAc_2_ and Man_9_GlcNAc_2_

**Figure 5:**
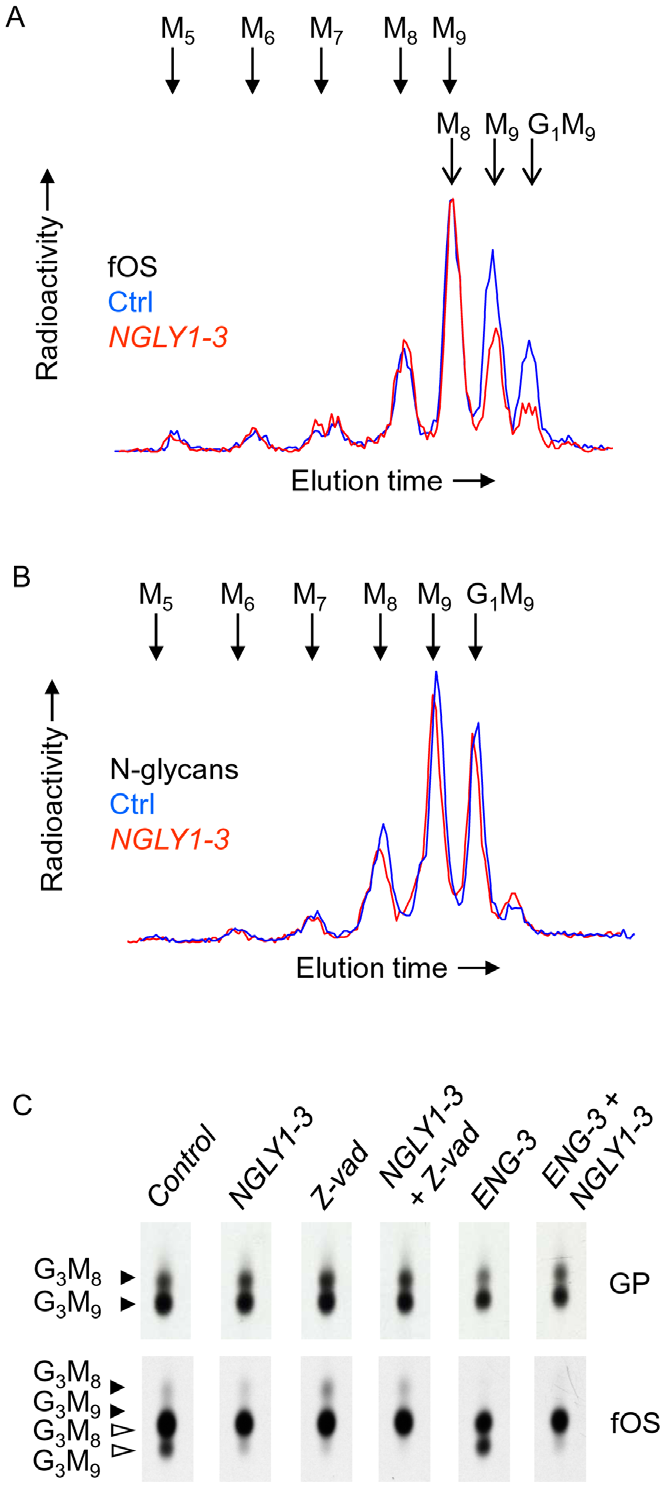
Examination of fOS, and N-glycans after NGLY1-3 or Z-vad mediated reduction of Ngly1p activity in control and castanospermine-treated cells. A. HepG2 cells were transfected with 25 pmoles of control siRNA (Ctrl), or 25 pmoles of *NGLY1-3*. Three days after transfection, cells were pulse-radiolabeled with [2-^3^H]mannose for 30 min and cellular EndoH-released [2-^3^H]*N*-glycans (*N*-glycans) and [2-^3^H]fOS (fOS) were prepared and analysed by HPLC. Whereas the whole fOS fractions were analysed, only 25% of the *N*-glycan fractions were examined by HPLC. The elution positions of standard oligosaccharides are indicated above the HPLC profiles and the abbreviations used are defined in the legend for Fig 2. B. HepG2 cells were transfected with 50 pmoles of control siRNA (Ctrl), or 25 pmoles each of *NGLY1-3* and 25 pmoles of control siRNA (NGLY), or 25 pmoles each of *ENG-3* and 25 pmoles of control siRNA (ENG), or 25 pmoles each of *NGLY1-3* and *ENG-3* (ENG + NGLY). Three days after transfection, cells were preincubated for 30 min with 2 mM castanospermine (CST), and where indicated, with 40 µM Z-vad-fmk (Z-vad). Cells were then pulse-radiolabeled with [2-^3^H]mannose for 30 min and cellular EndoH-released [2-^3^H]*N*-glycans (GP) and [2-^3^H]fOS (fOS) were prepared and examined by TLC. The scanned TLC lanes are from the same fluorograph, but due to uneven migration, the scans were aligned manually to facilitate interpretation of data. Closed and open arrowheads indicate the migration positions of components bearing one or two residues, respectively, of GlcNAc at their reducing end. The abbreviations are: G_3_M_9_; Glc_3_Man_9_GlcNAc_1-2_, G_3_M_8_; Glc_3_Man_8_GlcNAc_1-2_. C. These experiments were performed once.

To gain more insight into fOS generation during Ngly1p down regulation [2-^3^H]glycoproteins and [2-^3^H]fOS were examined under different experimental conditions indicated in Fig 5A and B. In this experiment, although Ngly1p down-regulation produced the expected effect on the distribution of fOS (Fig 5A), the *N*-glycan profile differed little from that derived from control cell incubations (Fig 5B). It was noted that EndoH-released *N*-glycans comprised 22.0%, 30.9% and 17.9% Glc_1_Man_9_GlcNAc, Man_9_GlcNAc and Man_8_GlcNAc, respectively, for the control RNAi-transfected cells, and 22.8%, 32.2% and 15.6%, respectively, for *NGLY1-3* transfected cells. As the Ngly1p-dependent fOSGN2 pool is enriched in Glc_1_Man_9_GlcNAc_2_ whose *N*-linked counterpart can interact with the lectins calnexin and calreticulin [2], we asked whether or not formation of the Ngly1p-dependent fOSGN2 pool required such *N*-glycan/lectin interactions. It is known that in order for glycoproteins to interact with calnexin and calreticulin, ER glucosidase-dependent trimming of triglucosylated *N*-glycans must occur [4]. Accordingly, the effect of ER glucosidase inhibition by castanospermine (CST) on Ngly1p-mediated fOS generation was investigated. Fig 5C demonstrates the presence of an Ngly1p-dependent pool of Glc_3_Man_9_GlcNAc_2_ and an Ngly1p-independent pool of Glc_3_Man_8_GlcNAc_2_ during ER glucosidase inhibition. In the same incubations it can be seen that the ratio of *N*-linked Glc_3_Man_9_GlcNAc_2_ to Glc_3_Man_8_GlcNAc_2_ remains similar under all the experimental conditions. Thus, an Ngly1p-dependent fOS pool was detected in the absence of monoglucosylated *N*-glycans. Furthermore, irrespective of glucosidase inhibition, the Ngly1p-dependent fOS pools are predominantly fully mannosylated whereas the Ngly1p-independent pools comprise oligosaccharides containing mainly 8 residues of mannose.

### Down regulation of Ngly1p leads to ∼30% inhibition of total fOS

**Figure 6:**
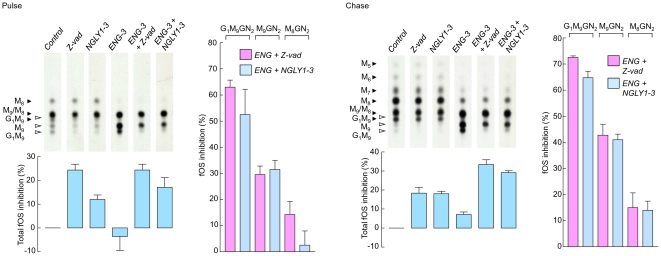
Quantitation of Ngly1p-dependent and -independent fOS pools in HepG2 cells. Cells were transfected with 50 pmoles of control siRNA (*Ctrl and Z-vad*), or 25 pmoles each of *NGLY1-3* and 25 pmoles of control siRNA (*NGLY*), or 25 pmoles each of *ENG-3* and 25 pmoles of control siRNA (*ENG*), or 25 pmoles each of *NGLY1-3* and *ENG-3* (*ENG + NGLY*). Three days after transfection, where indicated, cells were preincubated for 30 min with 40 µM Z-vad-fmk (Z-vad). Cells were then pulse-radiolabeled in either the absence or presence of Z-vad with [2-^3^H]mannose for 30 min (Pulse, left panels) or pulse-radiolabeled for 30 min prior to conducting a 1 h chase incubation (Chase, right panels). [2-^3^H]lipid-linked oligosaccharides, [2-^3^H]*N*-glycans released by EndoH from both cellular and medium glycoproteins and [2-^3^H]fOS were prepared and quantitated by scintillation counting. After summing the radioactivity associated with the above described fractions to generate total radioactivity incorporation into cells. [2-^3^H]fOS were examined by TLC. In order to take into account the differences in total incorporation of radiolabel into cells cultivated under the different conditions, the fraction of total fOS that was loaded onto the TLC was adjusted to take into account the ratio of the total cellular radioactivity for a given incubation to that recovered from the incubation incorporating least radioactivity. The migration positions of standard oligosaccharides are shown to the left of the chromatograms and the abbreviations used are as described in Fig 2. After elution of the oligosaccharide components from TLC plates and quantitation by scintillation counting, inhibition of total fOS appearance with respect to the control was calculated and is shown under the appropriate TLC lanes. The percentage inhibitions of individual oligosaccharides (Glc_1_Man_9_GlcNAc_2_: G_1_M_9_, Man_9_GlcNAc_2_: M_9_, Man_8_GlcNAc_2_: M_8_) observed in the *ENG + NGLY* and *ENG + Z-vad* conditions with respect to the *ENG* condition are shown to the right of the chromatograms. The scanned TLC lanes are from the same fluorograph, but due to uneven migration, the scans were aligned manually to facilitate interpretation of data. This experiment was repeated 4 times and the error bars represent the standard deviation.

Next the size of the Ngly1p-dependent fOS pool was calculated. Accordingly, pulse-chase studies were performed in control RNAi-, or *ENG-3*-transfected cells in which Ngly1p was either co-down regulated with *NGLY1-3*, or inhibited with Z-vad. In 30 min pulse radiolabeling incubations, Z-vad provokes about 25% total fOS inhibition whereas Ngly1p down regulation leads to about 12% fOS inhibition (Fig 6, Pulse, left panel). Down-regulation of Engase1p alone did not provoke a significant inhibition of total fOS, and comparing the Engase1p down regulation condition with the Engase1p and Ngly1p compromised conditions, similar total fOS inhibitions were noted (Fig 6, Pulse, left panel). In the latter conditions, where quantitation of Man_8_GlcNAc_2_ is more meaningful due to reduced fOSGN2-to-fOSGN conversion, substantial inhibitions of the appearance of fOS migrating as Glc_1_Man_9_GlcNAc_2_ and Man_9_GlcNAc_2_ were noted whereas lesser inhibition of Man_8_GlcNAc_2_ was observed (Fig 6, Pulse, right panel). Overall, however, *NGLY1-3-*, or Z-vad-mediated fOS inhibitions were similar irrespective of whether or not Engase1p was co-down regulated. In cells that were pulse radiolabeled and chased for 1 h (Fig 6, lower panels) the situation changed such that Engase1p down regulation alone caused a small (5–10%) but significant inhibition of total fOS generation (Fig 6, Chase, left panel). This inhibition of the appearance of fOS mediated by Engase1p down regulation alone was also reflected by increases in fOS inhibition observed when both Engase1p and Ngly1p are compromised compared to when the latter activity is reduced alone (Fig 6, Chase, left panel). Indeed, when both enzyme activities are reduced, ∼35% total fOS inhibition is noted in 1 h chase incubations. In addition it can be seen that, in the presence of *ENG-3*, the inhibitions of the appearance of fOS migrating as Glc_1_Man_9_GlcNAc_2_ Man_9_GlcNAc_2_ and Man_8_GlcNAc_2_ caused by Z-vad and *NGLY1-3* were higher than those noted for the pulse incubations (Fig 6, compare upper and lower right panels). Although Z-vad and *NGLY1-3* provoked small inhibitions of the appearance of Man_8_GlcNAc_2_, this inhibition may be overestimated because of incomplete Engase1p down regulation leading to contamination of this component with Glc_1_Man_9_GlcNAc and Man_9_GlcNAc whose appearance is inhibited by Z-vad and *NGLY1-3*. To summarise, taking into account that *ENG-3* itself causes a small but significant inhibition in the appearance of fOS, it can be concluded that the Ngly1p-dependent fOS pool corresponds to ∼30% of total fOS.

### Human Engase1p can generate fOS in Png1p-deficient yeast

**Figure 7:**
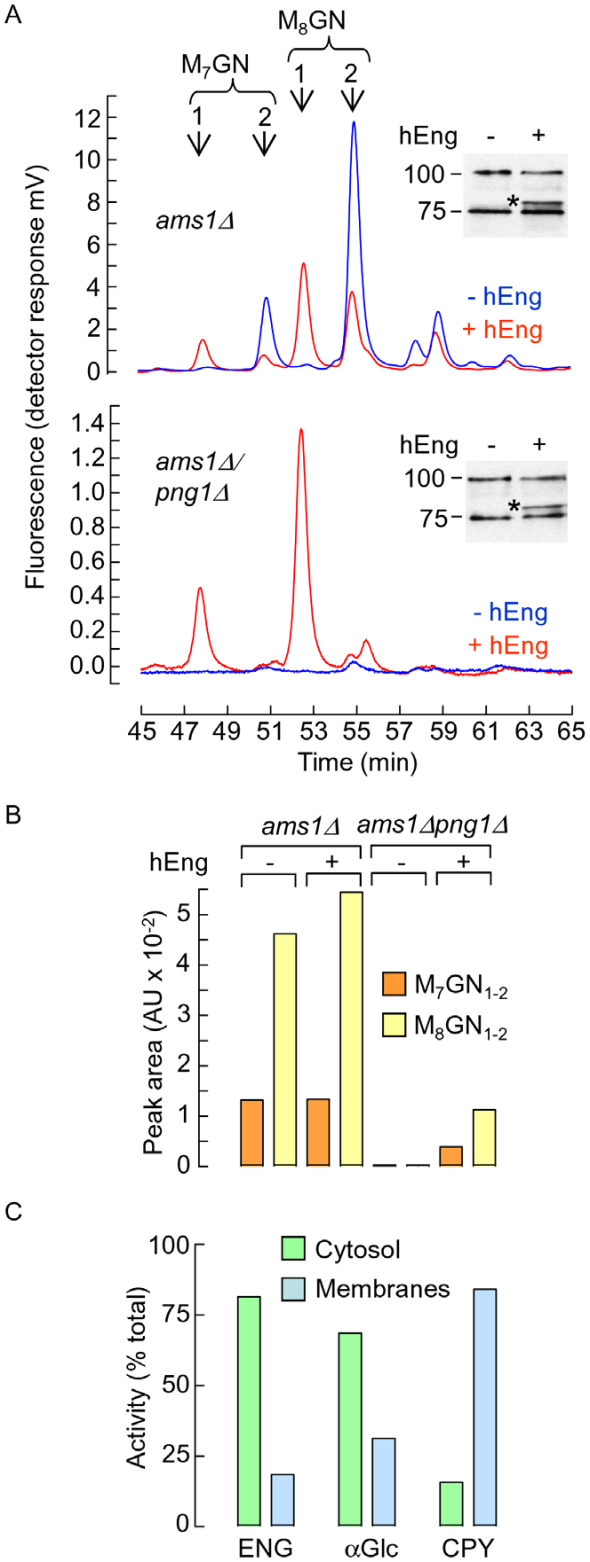
Examination of the deglycosylating role of human Engase1p in Png1p-deficient yeast cells. A. Obtention of the *S. cerevisiae ams1Δ* and *ams1Δpng1Δ* strains, deficient in the vacuolar mannosidase, Ams1p, and their transfection with an empty vector or a vector encoding Hist-tagged human Engase1p, is described in Materials and Methods. Cells were grown to mid log phase and fOS were extracted, purified and derivatised with 2-AP. Derivatised fOS (corresponding to 146, 92, 166 and 88 DU_600 nm_ for the *ams1Δ*, *ams1Δ^hENGASE^*, *ams1Δpng1Δ* and *ams1Δpng1Δ^hENGASE^* strains, respectively) were resolved by HPLC and detected using an on line fluorescence detector. Arrows indicate the elution times of fOS containing either a single (1) or two (2) residues of *N*-acetylglucosamine at their reducing termini. Insets. The *ams1Δ* (upper panel) and *ams1Δpng1Δ* (lower panel) strains transfected with the empty vector (− hEngase1p) or the vector encoding Hist-tagged human Engase1p (+ hEngase1p) were grown to mid log phase and cell extracts were probed with an anti Hist tag antibody after SDS PAGE by Western blot. Hist-tagged hEngase1p (*) has a molecular mass of 80 kDa. The migration position of 100 and 75 kDa markers are indicated to the left of the blots. B. After correction for the different quantities of starting material used for each yeast strain the areas under the HPLC peaks corresponding to Man_7_GlcNAc_2_ and Man_7_GlcNAc (M_7_GN_1-2_) were summed, as were those for Man_8_GlcNAc_2_ and Man_8_GlcNAc (M_8_GN_1-2_). C. Wild type cells were transfected with the vector encoding Hist-tagged human Engase1p, grown to mid log phase, converted into spheroplasts, and permeabilised as described in Materials and Methods. After centrifugation, cytosol and membrane fractions were assayed for Engase1p (ENG), cytosolic α-glucosidase (αGlc) and vacuolar carboxypeptidase Y (CPY) activities which have been expressed as a percentage of the sum of the cytosol and membrane values. This experiment was performed once.

To further understand the potential physiological role of Engase1p in glycoprotein deglycosylation, the capacity of this enzyme to generate fOS in yeast was examined. Although *S. cerevisiae* does possess cytosolic PNGase (Png1p, [20]) it does not contain an Engase1p homolog and previous studies indicated that fOSGN2 but not fOSGN are generated in this organism [21]. Accordingly, the *S. cerevisiae* Png1p null mutant (*png1Δ*) is a useful strain with which to study the role of human Engase1p (hEngase1p) in glycoprotein deglycosylation. Additionally, in order to facilitate interpretation of results, the gene encoding the vacuolar mannosidase, Ams1p, was deleted in the *png1Δ* strain because it is known that this mannosidase degrades fOSGN2. In fact, Ams1p expression is under glucose repression and the enzyme is first synthesised in the cytosol before being translocated into the vacuole [41]. Exponentially growing strains were harvested and fOS were extracted, purified and derivatised with the fluorophore 2-AP. HPLC analysis of the resulting mixtures indicates that whereas both the *ams1Δ* and *ams1Δpng1Δ* strains elaborate fOSGN2 containing mainly 7 and 8 residues of mannose, there is a ∼50 fold reduction of these components in the latter strain (Fig 7A, compare blue traces in upper and lower panels, and Fig 7B). In yeast, an *N*-glycan bearing 7 residues of mannose [42] that arises through the sequential actions of the mannosidases Mns1p and Html1p [43] is thought to be involved in Yos9p-targeting [42] of misfolded lumenal ER glycoproteins for proteasomal degradation in the cytosol. These results are reinforced by our previous findings demonstrating that whereas the Man_7_GlcNAc_2_ *N*-glycan can not be detected in total glycoprotein mixtures in the *ams1Δ* strain, the corresponding fOSGN2 is relatively abundant [21]. Upon transfection of the *ams1Δ* strain with Hist-tagged hEngase1p, about two thirds of total fOS are now recovered as fOSGN (Fig 7A, red trace in upper panel). However the total amounts of Man_7_GlcNAc_1-2_ generated in the *ams1Δ* and *ams1Δ^hENGASE^* strains are the same (Fig 7B). Although total Man_8_GlcNAc_1-2_ generated in the *ams1Δ^hENGASE^* strain appears about 15% greater than that recovered from the *ams1Δ* strain (Fig 7B) it is apparent that in the *ams1Δ^hENGASE^* strain the peak corresponding to Man_8_GlcNAc_2_ comprises several species thereby causing an over estimation of total Man_8_GlcNAc_1-2_ in this strain. Nevertheless, despite the absence of unambiguous evidence for a protein deglycosylating function of hEngase1p in the *ams1Δ* strain, when the *ams1Δpng1Δ* strain expresses hEngase1p, there is a clear generation of fOSGN species containing 7 and 8 residues of mannose (Fig 7A, red trace in lower panel) whose quantities represent ∼20% of that of their Png1p-generated fOSGN2 counterparts recovered from the *ams1Δ* strain (Fig 7B). Several observations indicate that in the *S. cerevisiae png1Δ* deletion mutant, hEngase1p can have a deglycosylating role similar to that of Png1p. First, the HPLC profile of Png1p-generated fOSGN2 observed in the *ams1Δ* strain (Fig 7A, blue trace in upper panel) is similar to that of the Engase1p-generated fOSGN generated in the *ams1Δpng1Δ^hENGASE^* strain (Fig 7A, red trace in lower panel). Second, *N*-glycans bearing 7 residues of mannose are thought to be specific to misfolded glycoproteins that are translocated from the ER into the cytosol. Third, subcellular fractionation studies indicate that hEngase1p is located in the cytosol of transfected yeast (Fig 7C). Nevertheless, as Engase1p expression levels are similar in the *ams1Δ^hENGASE^* and *ams1Δpng1Δ^hENGASE^* strains (Fig 7A, inserts) it can be concluded that, at least in a yeast background, hEngase1p is more effective at converting fOSGN2 into fOSGN than generating fOSGN from glycoproteins.

## Discussion

### The role of Engase1p in the conversion of fOSGN2 to fOSGN

Although previous studies on the substrate specificity of the cytosolic mannosidase (Man2c1p) demonstrated that its preferred substrates are fOSGN [37], the gene product(s) required for conversion of fOSGN2 into fOSGN in mammalian cells has not been clearly identified. Here, we demonstrate in a mammalian cell line that down regulation of ENGASE expression leads to: (i) a block in the appearance of fOSGN; (ii) a slowdown of both the disappearance of cytosolic fOSGN2 and the appearance of smaller MBC-situated fOSGN (< Man_7_GlcNAc) during chase incubations; (iii) increased steady state levels of cytosolic fOSGN2, and (iv) decreased steady state levels of several small cytosolic and MBC-situated fOSGN (< Man_5_GlcNAc). Accordingly, several lines of evidence demonstrate the importance of Engase1p in the conversion of fOSGN2 into fOSGN. Although the ‘leakiness’ of the block in fOSGN2 to fOSGN conversion observed in the presently reported RNAi studies may be attributable to only 70% down regulation of ENGASE mRNA, the possibility that, in addition to Engase1p, another enzyme is involved in this process cannot be excluded.

### The role of Engase1p in the subcellular trafficking of fOS

Next, down regulation of Engase1p and Man2c1p was undertaken in order to compare the roles of these two enzymes on fOS trafficking in HepG2 cells. Oligosaccharide structures recovered from different cellular compartments were examined after metabolic radiolabeling and chase incubations, and at steady state. The former studies performed in the presence of CCMA revealed that both Engase1p and Man2c1p are required for the efficient translocation of cytosolic fOS into MBC. Furthermore, it is demonstrated that both Engase1p and Man2c1p regulate fOS steady state levels in both the cytosol and MBC compartments of SLO-permeabilised HepG2 cells. HPLC profiles of the steady state levels of cytosolic fOS reported herein are similar to those reported for the cytosol of HepG2 cells [38]. Importantly, we show that as well as provoking an accumulation of large fOSGN, Man2c1p down-regulation causes a 90% reduction of the steady state level of linear Man_5_GlcNAc, one of the terminal digest products of Man2c1p. Accordingly, contrary to that seen in HEK-293 cells [17], the bulk of linear Man_5_GlcNAc is generated by Man2c1p in HepG2 cells. Engase1p down-regulation also reduces the steady state level of linear Man_5_GlcNAc, but this reduction is lesser in magnitude probably as the accumulations of fOSGN2 observed under these conditions are less pronounced than the accumulations of large fOSGN provoked by Man2c1p down-regulation. Reductions in total Man_5_GlcNAc steady state levels by either Engase1p or Man2c1p down-regulation are accompanied by reductions in Man_4-3_GlcNAc recovered predominantly from the MBC. The peaks co migrating as standard Man_4_GlcNAc, Man_5_GlcNAc and Man_6_GlcNAc (containing mainly Glc_1_Man_5_GlcNAc) are known to correspond to the limit digest products of Man2c1p action on different cytosolic polymannose structures in which the α1,3-, or A branch, is either missing its terminal mannose, intact, or is substituted with glucose, respectively [38]. The absence of the Man_3_GlcNAc structure in the cytosolic compartment along with its reduction in the MBC of *M2C1-1* and *ENG-3* transfected cells suggests that this structure originates from lysosomal trimming of cytosol-derived Man_4_GlcNAc and Man_5_GlcNAc after transport into lysosomes. Although these results agree well with the previously described biochemical characteristics of the lysosomal fOS transporter which is specific for partially demannosylated oligosaccharidic structures [12], it was consistently noted during pulse-chase studies performed in the absence or presence of CCMA that this reagent not only provoked major accumulations of small fOS in the MBC, but also caused less striking but clear MBC accumulations of larger fOS (See Fig 2B and C; compare upper and lower panels) known to be poor substrates for the lysosomal fOS transporter. Whether or not these larger structures are slowly transported into lysosomes remains to be established, but the use of 3-methyladenine, a well known inhibitor of autophagic sequestration, indicates that these structures may not be sequestered into the endomembrane system by the classical autophagy-lysosomal pathway (Chantret, I. and Moore, S. unpublished observations).

### Engase1p and Man2c1p are required for externalization of fOS in CCMA-treated HepG2 cells

We previously published that a small fraction of fOS can be found in the medium of CCMA-treated HepG2 cells [11]. Here, it is shown that when compared to intracellular cytosolic- or MBC-situated fOS species, the extracellular pool is enriched in smaller components indicating that either fOS are degraded in the medium or the externalization process is selective towards smaller components. Since the externalization process is not inhibited by BFA (data not shown), it is distinct from the classical Golgi apparatus-dependent secretion pathway. CCMA inhibits the vacuolar ATPase and perturbs vesicular trafficking including endosomal transport [44]. As well as modifying the intracellular distribution of lysosomal β-hexosaminidase [11], observations indicate that CCMA also causes a BFA-independent appearance of lysosomal β-hexosaminidase in the culture medium without accompanying cell damage as assessed by extracellular LDH levels (Chantret, I. and Moore, S. unpublished observations). In the light of these results, it can be hypothesised that perturbation/fusion of elements of the lysosome/endosome system allows secretion of lysosomal contents into the extracellular space. Whatever the mechanism underlying the appearance of fOS in the extracellular space of CCMA-treated HepG2 cells both Engase1p and Man2c1p are required for this process.

### Does Engase1p deglycosylate glycoproteins under physiological conditions?

This has proved a difficult question to answer. In a *C. elegans* ENGASE null mutant, an accumulation of fOSGN2 was noted to accompany a net reduction of total fOS [45]. This phenomenon was interpreted to be a consequence of inhibition of Ngly1p by high levels of fOSGN2 [46]. Here, we were unable to demonstrate an inhibition of the appearance of fOS in pulse-radiolabeled Engase1p compromised cells, but examination of fOS in 1 h chase incubations revealed a 5–10% inhibition of fOS mediated by Engase1p down regulation. It is unlikely that these results are due to inhibition of Ngly1p by fOSGN2, as the 6–11 fold increases of fOSGN2 steady state levels observed (Fig 3A and Table 1) are not likely to change between the pulse and chase periods. As this enzyme is more active toward fOS and glycopeptides than glycoproteins [27] it perhaps plays a role in the deglycosylation of a small pool of glycoproteins/glycopeptides after the bulk of fOS have been generated by other mechanisms. A deglycosylating role for Engase1p is reinforced by our demonstration that, *in vivo*, hEngase1p can liberate fOSGN in the *S.cerevisiae ams1Δpng1Δ* strain. Finally, glycoproteins that have been deglycosylated by an ENGase activity will possess residual *N*-linked GlcNAc residues, and in fact just such glycoproteins have recently been identified in cultured cells but at present it is not clear whether or not they were deglycosylated by an ENGase activity [47]. Accordingly, the importance of Engase1p in glycoprotein/glycopeptide deglycosylation under normal physiological conditions remains to be determined. Furthermore, under certain conditions of ER or cellular stress the deglycosylating roles Ngly1p and Engase1p may change.

### The role of Ngly1p in the generation of fOSGN2

In this study, we demonstrated the presence of a minor short NGLY1 transcript potentially encoding a C-terminally truncated protein. This transcript was identified in Caco 2 cells and in primary fibroblast cultures (Chantret, I. and Moore, S. unpublished observations) as well as HepG2 cells. Whether or not a protein is encoded by this short transcript remains to be established, but our results suggest that it is not involved in fOS generation. By contrast, our data clearly demonstrate a role for the major, full length, NGLY1 transcript in the generation of a limited pool of fOSGN2 that is enriched in Glc_1_Man_9_GlcNAc_2_ and Man_9_GlcNAc_2_ when compared to the total fOS pool. This relatively restricted role of Ngly1p on fOS generation in HepG2 cells was unexpected in view of previous work conducted on *S. cerevisiae* where Png1p, the yeast Ngly1p ortholog, is responsible for the generation of greater than 70% of all fOS in yeast [21]: see also Fig 7A and B where steady state fOS levels in the *ams1Δpng1Δ* strain are about 2% of that occurring in the *ams1Δ* strain. Any explanation of this weak effect of NGLY1 mRNA down regulation must take into account the fact that the PNGase inhibitor Z-vad-fmk yields the same result. Differences have also been observed between yeast and mammalian cells concerning the role of Ngly1p during degradation of ERAD substrates. Indeed, in the *png1Δ* strain an important slowing down or a total block of the degradation of ERAD substrates like CPY* [20] or ricin A [48] is observed. By contrast, studies in mammalian cells have favored a less important role of Ngly1p during ERAD. Thus, down-regulation of NGLY1 in cells stably transfected with siRNA duplexes [49] or with the use of Z-vad-fmk [40], did not dramatically modify the degradation rate of different misfolded glycoproteins which were known to be ERAD substrates. These data combined with results from this study, reinforce the idea that while Png1p plays a crucial role in the degradation of misfolded glycoproteins in yeast, the presence of an Engase1p in mammalian cells may allow deglycosylation of some glycoproteins and subsequent proteasomal degradation in the absence of Ngly1p. The extent of the Ngly1p-dependent pool has been difficult to determine, but a 30–40% inhibition of total fOS was obtained after 1h of chase when Ngly1p was co-down regulated with Engase1p. When Engase1p is down regulated it was noted that the Ngly1p-generated fOSGN2 pool comprised mainly Glc_1_Man_9_GlcNAc_2_ and Man_9_GlcNAc_2_ structures whereas the Ngly1p-independent pool comprised mainly Man_8_GlcNAc_2_. Likewise, when ER glucosidases are inhibited, Ngly1p down-regulation inhibited Glc_3_Man_9_GlcNAc_2_ generation whereas Glc_3_Man_8_GlcNAc_2_ generation was unaffected. The paucity of Man_8_GlcNAc_2_ structures in the Ngly1p-dependent fOS pool is intriguing as some ERAD pathways are thought to involve mannose trimming of the *N*-glycans of misfolded glycoproteins [50]. Whether or not these pathways are extensively solicited in unstressed mammalian cells is unknown. Perhaps the untrimmed nature of the Ngly1p-dependent fOSGN2 observed in this study (Glc_1-3_Man_9_GlcNAc_2_) reflects an early cotranslational pre-emptive ERAD mechanism thought to be able to clear proteins that are blocked in the transclocon [51]. Although it is not clear which if any of the presently described ERAD processes could be at the origin of the Ngly1p-generated oligosaccharide pool(s) observed in our study, the predominantly fully mannosylated status of these fOS differentiates them from the Ngly1p-independent fOSGN2 containing 8 residues of mannose. Some observations suggest that this latter fOS pool may originate from LLO: first, at present Ngly1p and Engase1p are the only two enzymes known to generate fOS from glycoproteins; second, *in vitro* experiments suggest that fOSGN2 derived from LLO in the lumen of the ER are rapidly processed to Man_8_GlcNAc_2_ before transport into the cytosol [10]. If LLO hydrolysis is the major source of fOS in normally cultured mammalian cells, the question that leads on from these results is: are fOS generated merely as a byproduct of LLO regulation or do they have some function in the cell as has been proposed for these structures in *Campylobacter jejuni* [52]?

To conclude, we describe Ngly1p-dependent and -independent fOSGN2 pools. Engase1p is able to release fOSGN from glycoproteins in *png1Δ* yeast strains, and some evidence is presented to indicate that it may have a deglycosylating role in HepG2 cells. We demonstrate that conversion of fOSGN2 to fOSGN, the first comitted step in the clearance of cytosolic fOS into lysosomes, is mediated by Engase1p. Finally, we demonstrate that in HepG2 cells the bulk of fOS are not generated by either Ngly1p or Engase1p suggesting the presence of other protein deglycosylating enzymes or substantial generation of fOS from LLO. The mechanisms underlying the generation of the various fOS pools in mammalian cells certainly merit further study because they reflect key control processes during protein *N*-glycosylation, quality control and ERAD.

## Supporting Information

Table S1 Primers used in this study
(0.03 MB XLS)

Figure S1 Inhibition of ENG1 gene expression induced by siRNA in HepG2 cells - Cells were transiently transfected with 1, 10 and 100 pmoles of either negative control RNAi duplexes or 3 sets of ENG1 RNAi duplexes (ENG-1, ENG-2 and ENG-3). Total mRNA were extracted 3 days later and ENGASE mRNA levels were quantitated by QPCR as described in Material and Methods.
(2.88 MB TIF)

Figure S2 Effects of the inhibition of Man2C1 gene expression on fOS metabolism - (A) Kinetics of inhibition of Man2C1 transcripts by 3 siRNA duplexes. HepG2 cells were transiently transfected with 25 pmoles of 3 different short interfering RNA sequences (M2C1-1, M2C1-2 and M2C1-3) targetting the cytosolic Man2C1 mannosidase. 1, 2 and 3 days post-transfection, cDNA was prepared. Quantitative PCR was used to estimate changes in mRNA levels of the Golgi Man2A1, lysosomal Man2B1 and cytosolic Man2C1 mannosidases relative to those observed in cells transfected with a control interfering RNA sequence. (B) Cells were transfected with either negative control RNAi duplexes (Ctrl) or Man2C1 RNAi duplexes (M2C1-1, M2C1-2 and M2C1-3) 2 days before pulse-radiolabeling with [2-3H]mannose for 30 min. Subsequent to pulse or 4 h of chase incubations, fOS were extracted from cells and chase media as described in Material and Methods. Purified fOS were then resolved by thin layer chromatography on silica-coated plates. The migration positions of standard oligosaccharides are shown to the left of the chromatographs and the abbreviations associated with the open arrowheads are: G1M9, Glc1Man9GlcNAc2 ; M9, Man9GlcNAc2 and M8, Man8GlcNAc2. Those associated with the closed arrowheads are: G1M9, GlcMan9GlcNAc; M9, Man9GlcNAc; M8, Man8GlcNAc; M7, Man7GlcNAc; M6, Man6GlcNAc; G1M5, GlcMan5GlcNAc; and M5, Man5GlcNAc. (C) Negative control RNAi duplexes (Ctrl) or RNAi duplexes targeting Man2c1 (M2C1-1, M2C1-2 and M2C1-3) transfected cells were pulse-radiolabeled with [2-3H]mannose, and where indicated, were pretreated with 100 µM swainsonine (Ctrl+SW). Subsequent to performing 6 h chase incubations in either the presence or absence of SW, glycopeptides were prepared from both cells and media. Con A-Sepharose affinity chromatography was used to quantitate complex-, hybrid- and polymannose-type glycopeptides as described in Materials and Methods section. SW was used as a positive control in order to verify that the Con A-Sepharose affinity chromatography was able to detect changes in protein glycosylation induced by inhibiting Golgi mannosidase II.
(9.55 MB TIF)
