# Supplementary figures and images for: Identification of Roles for Peptide: N-Glycanase and Endo-β-N-Acetylglucosaminidase (Engase1p) during Protein N-Glycosylation in Human HepG2 Cells
Source: PLoS One. 2010 Jul 23;5(7):e11734. doi: 10.1371/journal.pone.0011734 (PMC2909182; doi:10.1371/journal.pone.0011734)

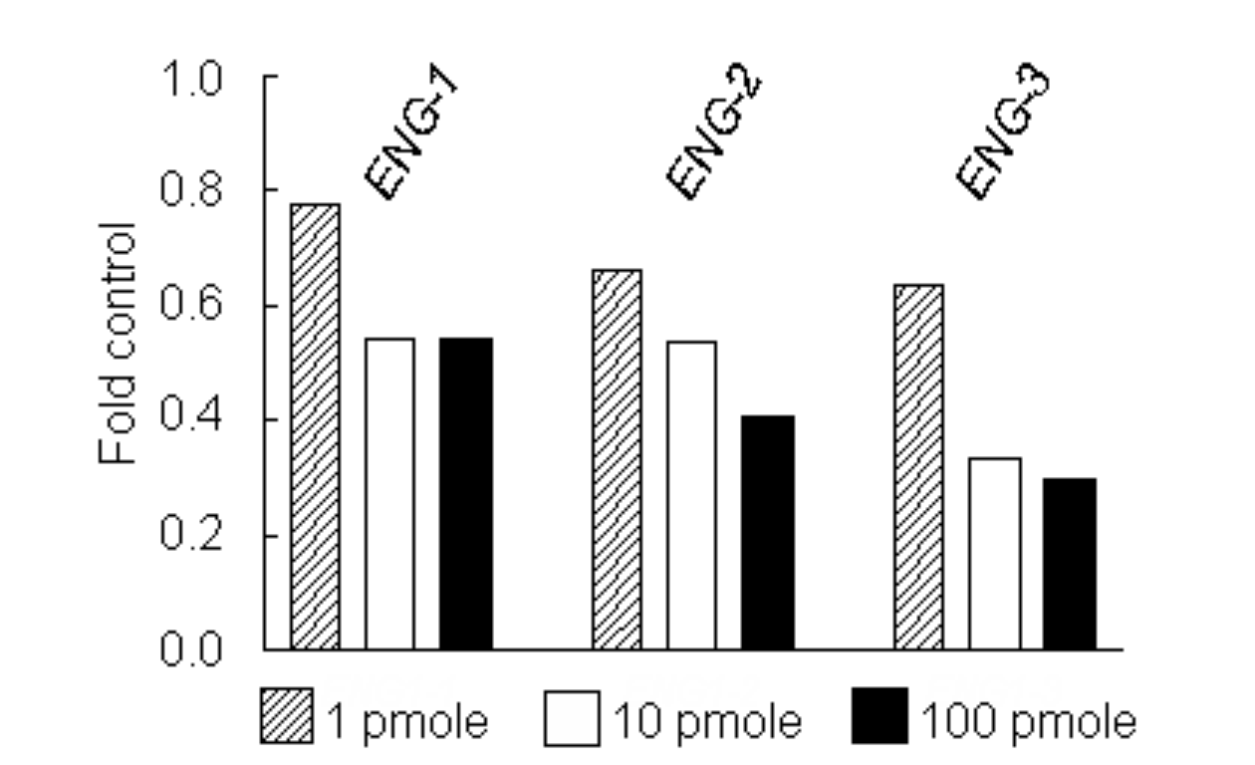

Supplement: Figure S1 — Inhibition of ENG1 gene expression induced by siRNA in HepG2 cells - Cells were transiently transfected with 1, 10 and 100 pmoles of either negative control RNAi duplexes or 3 sets of ENG1 RNAi duplexes (ENG-1, ENG-2 and ENG-3). Total mRNA were extracted 3 days later and ENGASE mRNA levels were quantitated by QPCR as described in Material and Methods. (2.88 MB TIF) [file pone.0011734.s002.tif]

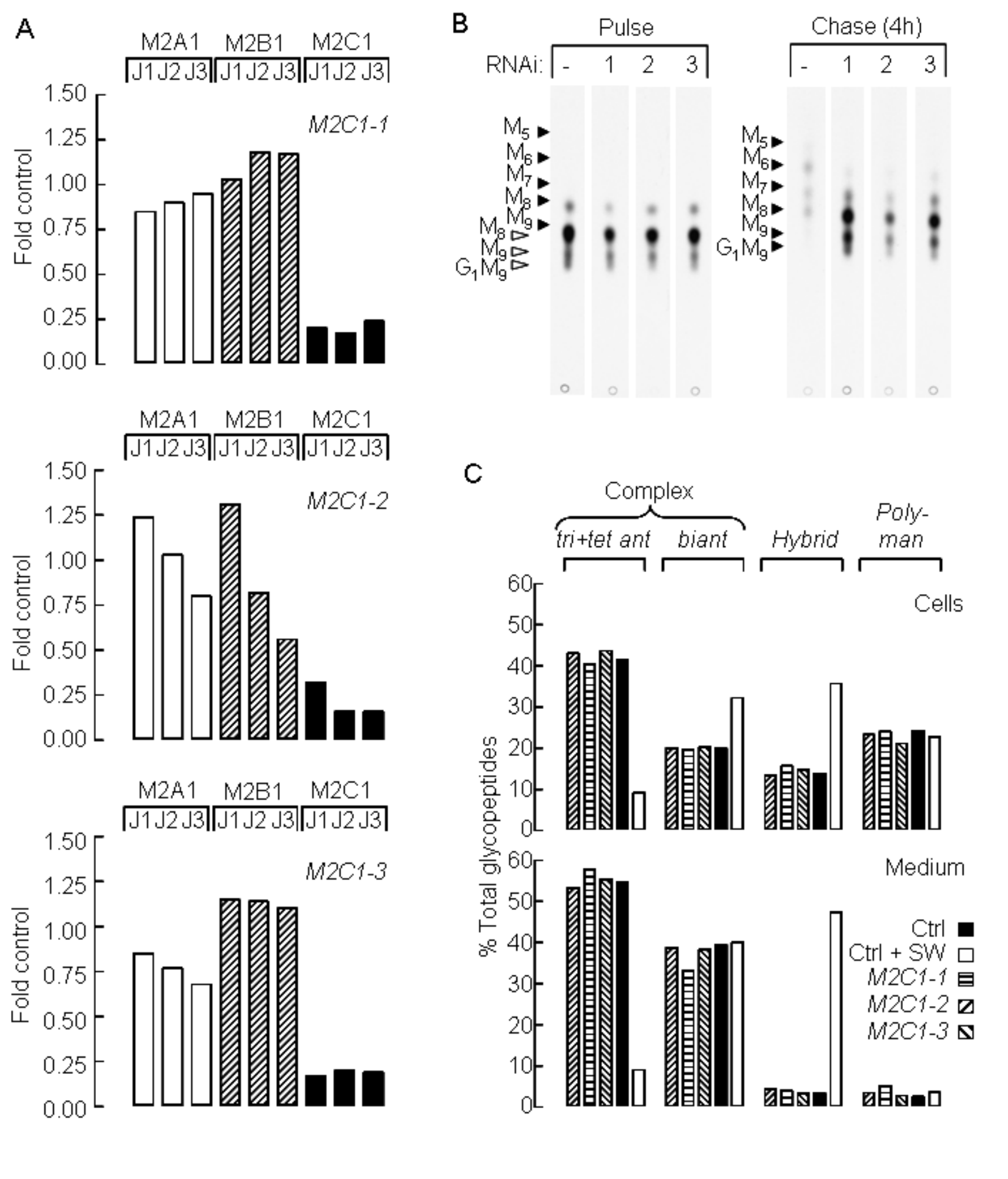

Supplement: Figure S2 — Effects of the inhibition of Man2C1 gene expression on fOS metabolism - (A) Kinetics of inhibition of Man2C1 transcripts by 3 siRNA duplexes. HepG2 cells were transiently transfected with 25 pmoles of 3 different short interfering RNA sequences (M2C1-1, M2C1-2 and M2C1-3) targetting the cytosolic Man2C1 mannosidase. 1, 2 and 3 days post-transfection, cDNA was prepared. Quantitative PCR was used to estimate changes in mRNA levels of the Golgi Man2A1, lysosomal Man2B1 and cytosolic Man2C1 mannosidases relative to those observed in cells transfected with a control interfering RNA sequence. (B) Cells were transfected with either negative control RNAi duplexes (Ctrl) or Man2C1 RNAi duplexes (M2C1-1, M2C1-2 and M2C1-3) 2 days before pulse-radiolabeling with [2-3H]mannose for 30 min. Subsequent to pulse or 4 h of chase incubations, fOS were extracted from cells and chase media as described in Material and Methods. Purified fOS were then resolved by thin layer chromatography on silica-coated plates. The migration positions of standard oligosaccharides are shown to the left of the chromatographs and the abbreviations associated with the open arrowheads are: G1M9, Glc1Man9GlcNAc2 ; M9, Man9GlcNAc2 and M8, Man8GlcNAc2. Those associated with the closed arrowheads are: G1M9, GlcMan9GlcNAc; M9, Man9GlcNAc; M8, Man8GlcNAc; M7, Man7GlcNAc; M6, Man6GlcNAc; G1M5, GlcMan5GlcNAc; and M5, Man5GlcNAc. (C) Negative control RNAi duplexes (Ctrl) or RNAi duplexes targeting Man2c1 (M2C1-1, M2C1-2 and M2C1-3) transfected cells were pulse-radiolabeled with [2-3H]mannose, and where indicated, were pretreated with 100 µM swainsonine (Ctrl+SW). Subsequent to performing 6 h chase incubations in either the presence or absence of SW, glycopeptides were prepared from both cells and media. Con A-Sepharose affinity chromatography was used to quantitate complex-, hybrid- and polymannose-type glycopeptides as described in Materials and Methods section. SW was used as a positive control in order to verify [file pone.0011734.s003.tif]
